# Modeling psychopathology in high-dimensional vector space using the high-dimensional symptom space (HDSS) model can operationalize precision psychiatry in US adolescents

**DOI:** 10.1038/s41598-025-18975-y

**Published:** 2025-10-08

**Authors:** Marcus G. Wild, Rebecca A. Cutler

**Affiliations:** 1 United States Department of Veterans Affairs, VHA VISN 17 Center of Excellence for Research on Returning War Veterans, 4800 Memorial Drive (151C), Waco, TX 76711 USA; 2 University of Texas at Austin, Austin, TX USA

**Keywords:** High-dimensional space, Psychopathology models, Precision psychiatry, HiTOP, P-factor, Psychology, Human behaviour, Computational models

## Abstract

Symptoms of psychopathology vary across people, limiting inferences about origins and treatments of disorders for any one person. The high-dimensional symptom space (HDSS) model offers a novel framework for understanding psychopathology by representing symptoms as vectors within a multidimensional space. Unlike traditional categorical and dimensional models, HDSS uses geometric distances to empirically characterize a person’s unique experience of symptoms, with the option to integrate social and cultural factors for more precise, personalized treatments. Using data from the adolescent brain and cognitive development (ABCD) study, we demonstrate that HDSS preserves individual specificity, effectively captures dynamic trajectories of psychological distress, and accommodates clinical heterogeneity. Results indicate that HDSS distances correspond to symptom severity and capture nuanced patterns of psychological distress over time, offering a comprehensive and individualized understanding of psychopathology. This model allows for a person-centered understanding of psychopathology, highlighting unique symptom patterns and their evolution over time. HDSS represents a significant advancement in personalized psychological care, providing a data-driven framework for understanding psychopathology symptoms, and implementing effective interventions.

**Supplementary Information:**

The online version contains supplementary material available at 10.1038/s41598-025-18975-y.

## Introduction

From the earliest theories of psychopathology, attempts to model and understand human psychological distress have sought to carve nature at its joints, relying on increasingly complicated heuristics of human experience to derive categories and dimensions of disorder. A central issue of this approach is that every person’s experience of psychopathology defies clean classification, despite shared features with others with similar experiences^1–3^. Further, current models waste patient data that could inform person-level etiology and treatment. The premise of this paper is that modeling a person and their distress relative to others may move the field closer to a culturally meaningful and individually relevant modeling of psychopathology. The article presents a high-dimensional symptom space (HDSS) model of psychopathology that overcomes problems with classical categories of psychopathology and approaches based on dimensional reduction. Through a brief review of prior approaches to modeling psychopathology followed by a demonstration of the HDSS in a large representative sample of US adolescents, we argue that the HDSS model is an improvement on existing models of psychopathology that (1) allows for the deduction of symptom clusters empirically using geometric distances, (2) is responsive to the interactions among social and cultural information that inform normative psychological experience, and (3) enables the design of precise and personalized treatments to address those symptom clusters.

### Categories and similarity in psychopathology

A central challenge in applying psychological theory to practice is identifying a taxonomy of disorders that facilitates effective treatment. Traditionally, this taxonomy has relied on a medical model with discrete disorder categories that dovetail with the International Classification of Diseases (ICD). Similarity is a central concept in the process of developing a taxonomy. Shared features (i.e., similar symptoms) form the basis of diagnostic categories starting with the first Diagnostic and Statistical Manual of Mental Disorders (DSM)^4^. The debate around categories of psychopathology derived from expert consensus on feature similarity and criticisms of the DSM have existed since its inception^5–7^. Chief among these concerns is that an effective categorization system should maximize within-category similarity and minimize between-category similarity^8^. However, when the same criteria are used for multiple disorders, the between-category similarity is increased, reducing the likelihood of accurate classification. This overlap—due to common symptoms across categories of psychopathology—results in a lack of consistent diagnosis, high prevalence of comorbidity, and a large number of transdiagnostic symptoms^9–12^. These are all indications that the current models for conceptualizing psychopathology are not adequately capturing the complex interactions among symptoms involved in modal psychological distress.

Further, some psychological symptoms are more transdiagnostic and more frequently clustered than others. Irritability, sleep disturbance, and social deficit are three examples of non-specific symptoms that are found in multiple discrete categories of disorder (e.g., major depressive disorder (MDD), posttraumatic stress disorder (PTSD), and autism spectrum disorder (ASD)). Further, physiological correlates, including shared genomic, metabolomic, or proteomic profiles, can differentiate people with psychopathology from those without, but cannot distinguish specific disorders^13–17^. As a result, comorbidity is highly prevalent and accurate person-level prediction of discrete disorder diagnosis remains elusive^11,18,19^.

Finally, psychological disorders are socially and culturally derived, and the rules for what constitutes “disorder” change over time and space^20,21^. Moreover, evidence suggests that socio-economic factors are the most potent predictors of psychopathology development^22^. As a result, modern models of psychopathology must incorporate cultural and socio-economic information^23^.

### Dimensions

Current trends in clinical psychological science are shifting from discrete categories to dimensions of psychological distress^23^. The limits of categorical approaches motivated the creation of the Research Domain Criteria (RDoC) in the 2010s to focus on dimensions of psychopathology at multiple levels of analysis^24^. The prevailing taxonomy that arose from RDoC—the hierarchical taxonomy of psychopathology (HiTOP)— uses a bottom-up approach to characterize symptom dimensions and iteratively cluster and reduce them to understand higher-order organization of psychopathology^2,3^. HiTOP addresses the severe information loss imposed by a categorical approach, using a diversity of methods including latent variable models and network approaches^2,25,26^. The improved ecological validity of HiTOP is reflected in clinicians’ preference for dimensional theories of psychopathology and their endorsement of greater clinical utility in HiTOP-based conceptualizations^41^. However, a comprehensive multidimensional model of psychopathology has not yet been achieved^2,3,27–31^.

**Fig. 1:**
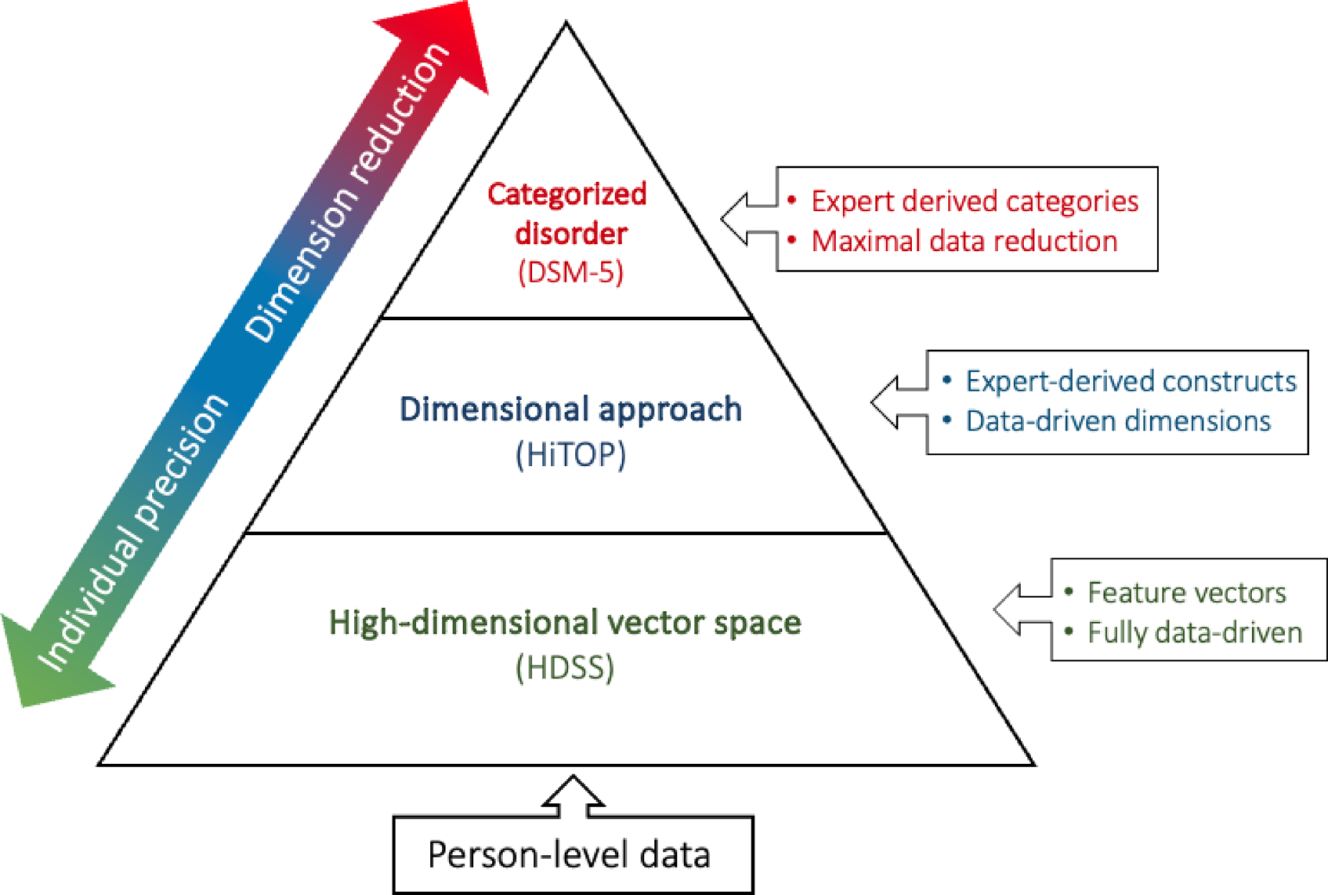
Schematic depicting conceptual frameworks from person to category level. The relative benefits and limitations of categorical disorders, dimensional approaches (e.g., HiTOP), and high-dimensional vector space approaches to modeling psychopathology.

Dimensional approaches such as HiTOP are fundamentally dimension reduction approaches, where a large number of features (i.e., high-dimensional space) are reduced to a smaller number of dimensions and clustered to create categories (Fig. 1)^32^. This method faces similar challenges to classical categorization, requiring maximization of within-cluster similarity and minimization of between-cluster similarity. Recent calls have advocated integrating top-down and bottom-up processes to develop more effective psychological interventions^33–35^. Craske and colleagues, for instance, have emphasized the need to integrate data from across diverse sciences to create more precise and effective diagnostic and treatment approaches^34^. Despite these efforts, current models do not adequately explain how psychopathology emerges from variable symptomatology^25^. Wilshire and colleagues note that a significant challenge is that models of psychopathology are based on symptoms that are themselves complex constructs^25^. While symptoms are still categories, they represent a much more specific and discernable experience. This is reflected in the reliability and validity of symptom measures, while validity of psychiatric diagnoses have not even been empirically established^36^.

The high-dimensional symptom space model (HDSS) allows for responses to individual items to drive characterization of psychopathology. As a representational symptom space that combines the unique set of experiences of one person to define a vector through that space, HDSS is fully informed by a person’s data and does not reduce dimensions. In this way, the vector is a precise and person-specific representation of a person’s experience, where dimension-reduction approaches (e.g., Hi-TOP) represent the relations among constructs, rather than people (see Fig. 1).

### The high-dimensional symptom space (HDSS) model of psychopathology

The issue of empirically modeling complex interactions among dimensions of features is not unique to clinical psychology. Computational neuroscience and genetics have pioneered approaches for capturing such high-dimensional interactions with multidimensional vector space models^37–39^. Computational models from cognitive neuroscience have been proposed to enhance understanding and modeling of psychological disorders^40,41^ with vector space models specifically used to understand psychological variation^42^emotion science theory, and cross-cultural variation of emotion^43,44^. Further, vector space models have been proposed as a means of modeling all human disease, supported by compelling recent evidence from the UK Biobank^45^.

HDSS is the first attempt to apply high dimensional vector space models to understand person-level experiences of psychopathology. The HDSS model is constructed from symptom-relevant features and is populated by vectors that represent people, permitting comparisons based on similarity between people rather than categories or dimensions. Similarity is represented as the distance between two vectors in the shared feature space. Applied to psychopathological symptoms, pairwise comparisons capture the dynamics of symptom space by representing each person as a unique combination of symptoms following a unique trajectory in symptom space.

For example, the similarity between symptom feature A (e.g., chronic pain intensity) and feature B (e.g., fear of pain) may be different depending on the context in which the symptoms are experienced. Chronic pain intensity may exist without fear of pain for some people, while others experience them together. Chronic pain and fear of pain may also be important for the same person after a surgery (context A), but not before (context B). The HDSS model quantitatively captures the dynamics both between and within people over time.

#### Navigating socially and culturally informed psychopathology space

**Fig. 2:**
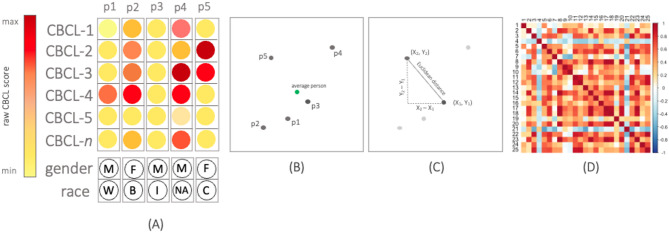
Visualizing psychopathology at the person level in HDSS. (**A**) Schematic of adolescents’ child behavioral checklist (CBCL) item response (i.e., CBCL-1 for item 1) and demographics vectors based on raw CBCL score (**B**) Adolescents projected into common space using multidimensional scaling (MDS) (**C**) Psychopathology at the person level can be calculated with geometric distances (e.g., Euclidean), based on their relative position in space (**D**) Pairwise correlation matrices show the similarity of psychopathology vectors between adolescents, hotter colors represent more similarity between to adolescents. Note, ‘p1’ through ‘p5’ refer to individual people, and X and Y in part C refer to coordinates in Euclidean space.

Conceptualizing psychopathology space as a landscape provides an intuitive way of understanding the possibilities of HDSS. Just as diverse terrains shape a physical landscape, the arrangement and distribution of symptoms in psychopathology space creates regions, clusters, and pathways. We can think of a patient’s trajectory through psychopathology space as a path or route (Fig. 2). Like moving from one location to another in a physical space, patients move through psychopathology “space” as their symptoms change over time. We can visualize the path they take as they navigate the space, considering factors like the order in which symptoms appear, their severity, and their changes over time. Crucially, this approach can include social and cultural data, offering a more comprehensive view of the patient’s journey. Transitions and jumps in psychopathology space may indicate significant shifts in symptoms, akin to changing environmental contexts in physical space. Identifying these transitions provides a unique way to address contributing factors, such as changes in treatment, life events, or underlying mechanisms. Another significant advantage of HDSS over standard measures is the ability to store and visualize information over time (Supplemental Figs. 2 and 3). In this way, we can capture the evolution of a person’s psychological health.

#### Prediction of person-level trajectories through symptom space

The HDSS model of psychopathology offers the advantage of not only describing existing data but also predicting future data. A crucial issue in psychopathology research has been the difficulties in predicting progression of disorder due to the highly dynamic course of psychological distress^10^. Attempts to address this issue from a HiTOP perspective have used computational models to accommodate high levels of heterogeneity in psychiatric genetics^46^ and been applied to human social features to explain the relations among socially-mediated psychiatric symptoms^47^. Further, deep learning approaches have shown promise in developing cross-diagnostic predictions of psychological disorders using multiple levels of data (e.g., genetics, age, psychological disorder diagnosis)^11,19,48^. However, these approaches do not provide person-level, prospective prediction of psychopathology courses, nor have they used individual profiles of distress, rather than categories, as units of analysis. The HDSS model addresses this gap by offering person-level predictions of psychopathology over time.

Motivated by these gaps in the current literature and the call and conceptual model offered by Craske and colleagues^34^ this paper aims to model psychological symptoms as vectors in multidimensional space using the HDSS model. We use the fourth wave data release from the Adolescent Brain and Cognitive Development (ABCD) study as an example dataset to demonstrate the benefits of the HDSS model and compare it to DSM-5 categories and HiTOP dimensions. The specific comparisons made are based on how well each approach (1) captures between and within category similarity, (2) preserves person-level specificity, and (3) represents severity of psychological distress.

## Methods

### Participants

Data from 11,876 adolescents from the Adolescent Brain and Cognitive Development (ABCD) study cohort were used to create the models (www.ABCDstudy.org*).* For a more detailed description of the cohort, methods, and considerations of the ABCD study, see previously published examinations^49–52^. All child participants provided assent to participate in the ABCD study and all parents provided their written informed consent. This work utilized the fully de-identified ABCD Data Release 4.0, which has been determined by the National Institutes of Health to not be human subjects research. The current study used a subset of the participants to include only those with complete data at all four of the annual timepoints (*n* = 5955).

### Measures

The Child Behavior Checklist (CBCL) was used for adolescent data^53^. The CBCL consists of 119 questions completed by the child’s parent, regarding the child’s behavior and characteristics. Items are scored on a scale of 0 (not at all), 1 (somewhat true), or 2 (very true). The CBCL can be scored to yield dimensional psychopathology subscale scores for five dimensions: total problems, externalizing symptoms, internalizing symptoms, attention problems, and somatic symptoms. For the purposes of the analyses in this paper, we used raw scores for each of the items. To calculate DSM-5 diagnoses, adolescents were considered to meet criteria for a disorder if their T score for that scale of the CBCL was 65 or greater, in line with scoring recommendations for the scale.

Demographic features were also included in analyses. Specific features included parent-reported race, gender, and age.

### Data analysis

#### Creating a multidimensional psychopathology space

We created a 119-dimensional CBCL psychopathology space, where each dimension represents an item response in the CBCL assessment. A person’s position in psychopathology space accurately captures their set of responses during a single assessment. To include demographic features, we iteratively added additional dimensions for race, parent-reported gender, and age. The relational structure between participants was calculated as a pairwise distance matrix, resulting in a single numeric value for the Euclidean distance between each response vector and all other response vectors. The Euclidean distance is the length of a single line between two vectors, $\:i$ (e.g., person 1) and $\:j$ (e.g., person 2), on an *n*-dimensional cartesian plane:

*d*_ij_ = $$\:\sqrt{({i}_{1}-\:{j}_{1}{)}^{2}\:+\:({i}_{2}-\:{j}_{2}{)}^{2}\:+\:\dots\:\:+\:({i}_{n}\:-\:{j}_{n}{)}^{2}\:\:}$$.

The distance, $\:{d}_{ij}$ represents the dissimilarity between two vectors, where higher values indicate a larger difference in responses, and identical responses have a distance of 0. These two vectors could be any feature set, such as the symptom and demographic features of two people, or the average vector of a group and a group member’s vector. Euclidean distance was chosen due to its simplicity in capturing the concept of distances between two vectors in a high-dimensional space and the fact that we used a single measure of psychopathology, meaning that each symptom measured was relevant despite any covariance with other symptoms, so we did not want to artificially downweight features. Other distance measures (e.g., Mahalanobis distance) can account for duplicate features and other sources of covariance. These measures were not used in the current study but could be beneficial in future iterations of HDSS. To visualize the data, we applied Multidimensional scaling (MDS); a dimensionality reduction technique that preserves the dissimilarities between vectors and allows us to represent the 122 space (119-dimensional CBCL subscale + race, age & gender) in just 2-dimensions. All elements were normalized to have a mean of 0 and a standard deviation of 1 so that each feature was equally weighted and contributed a single dimension to the space. The resulting symptom space was constructed independently of any diagnostic framework. To examine how clinical patterns are structured within it, we applied diagnostic groupings after constructing the space using two approaches: (1) DSM-5 disorder categories, and (2) HiTOP dimensions, using total scores from five CBCL T-scores; total problems, externalizing symptoms, internalizing symptoms, attention problems, and somatic symptoms. These classifications were then used to explore spatial clustering and trajectory patterns within the shared HDSS.

#### Centroid analysis

In order to compare a person’s position in space to a common referent, we first averaged the Child Behavior Checklist (CBCL) and demographic information of the 5955 people with all four data points to create a *‘average person vector’* for the ABCD dataset. This allowed us to conduct a centroid analysis; a data-driven way to calculate distances from a single reference point. We then calculated the Euclidean distance of each person, at each of the four timepoints, from the average vector. To help visualize the difference in distance from the centroid across disorders, we randomly generated a sample of 30 adolescents for each disorder to capture disorder-specific patterns and depicted these in rose diagrams.

#### Transition through space

Representing a person’s assessment trajectory within a single, standardized space has the benefit of capturing their current psychopathology relative to four years of assessment history. The CBCL psychopathology space using HDSS allows us to capture not only the relationship between people at one point in time, but also the relationship between one person across multiple time points. We can place CBCL response vectors from each of the four timepoints into a single standardized psychopathology space to capture a person’s trajectory based on this assessment. If there was little change in psychopathology symptoms or severity, a person’s responses would be similar to the previous year, and they would occupy a nearby location in space. However, as their patterns of responses change, their distance in psychopathology space increases. We also separated adolescents based on whether they met likely criteria for a diagnosis or not, based on a T score of 65 or greater for either any of the DSM-5 category dimensions (DSM approach) or the total problem dimension (dimensional approach). We then tracked their location in HDSS over the four available timepoints to see if adolescents without psychopathology who developed psychopathology moved closer to others with psychopathology, and vice versa, over time. Finally, a logistic regression classifier was trained on the first two HDSS dimensions to predict clinical status based on CBCL Total Problems scores (T > 64). The resulting decision boundary, defined by a probability threshold of 0.5, was used to visualize the division between clinically significant and non-clinically significant regions of psychopathology symptom space (Fig. 4).

#### DSM-5 categories and HiTOP dimensions in HDSS

To understand how the DSM-5 category or HiTOP dimension would be represented in MDS at baseline, we calculated the similarity of adolescents within a diagnostic category (e.g., the average Euclidean distance between each person diagnosed with the same disorder) or HiTOP dimension. We compared that to the similarity between different diagnostic categories (e.g., the Euclidean distance between people across two disorders) and dimensions. If adolescents with the same diagnosis or elevated in the same dimension were more similar, we would expect the within category distances to be significantly lower than the between category distances.

#### Clinical significance in HDSS

To test the idea that measures of Euclidean distance reflect clinical significance, we fit a linear regression model of Euclidean distance from the average person vector using CBCL item measures and the number of clinical disorders. While the space is generated from the CBCL and demographic items, as noted above, distance from the average person vector preserves all the information about each symptom endorsed and may therefore not scale precisely with number of disorders or dimensions endorsed. For example, those with a large number of symptoms endorsed may not meet criteria for any specific disorder or score highly on a dimension of psychopathology, so their distance from the average vector may be more informative than a diagnosis, a dimension, or a total score.

## Results

### Person-level psychopathology in HDSS via centroid analysis

**Fig. 3:**
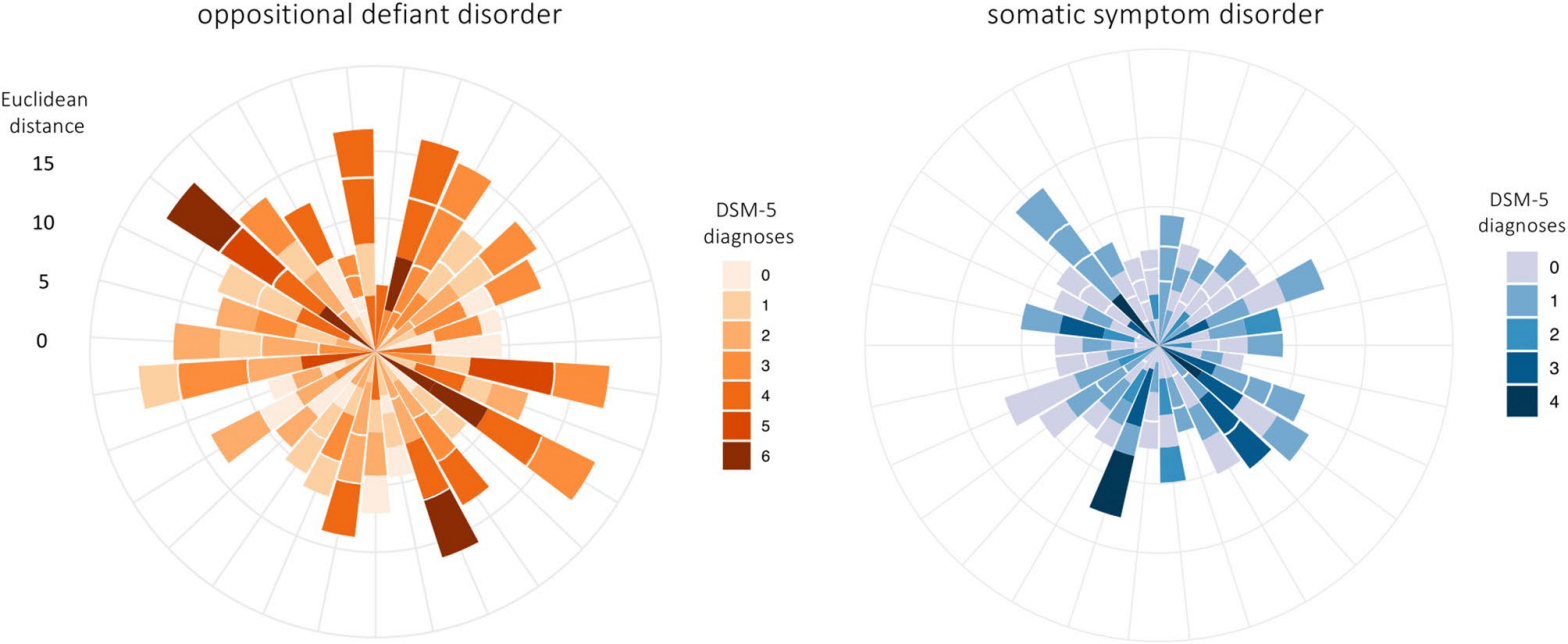
Centroid analysis: Rose plots show individual distance from the average person vector across four timepoints for adolescents with oppositional defiant disorder (left) and somatic symptom disorder (right). Each bar represents one person, and each segment reflects their Euclidean distance from the average vector at a given timepoint. Segment length changes across time indicate movement through symptom space: a shorter segment following a longer one reflects movement toward the average person vector (improvement), while a longer segment reflects divergence. Light-dark color axis indicates the number of DSM-5 diagnoses at each timepoint.

In Fig. 3 we highlight the variability between people based on disorder category using rose diagrams for oppositional defiant disorder and somatic symptom disorder (see Supplementary Fig. 1 for additional disorders). We chose these two disorders as examples for their lack of overlapping symptoms. Importantly, our average vector was not zero, meaning that the average person does not show the absence of all symptoms. Figure 3 captures some interesting disorder-specific patterns. For example, adolescents with oppositional defiant disorder were more likely to reach clinical significance for up to six disorders than adolescents with somatic symptom disorder^54^. Additionally, disorders differ in the distance from the average vector (e.g., people with somatic symptom disorder are closer to the average vector than people with oppositional defiant disorder), perhaps reflecting a more normative experience.

### Within-person trajectories over time in HDSS

**Fig. 4:**
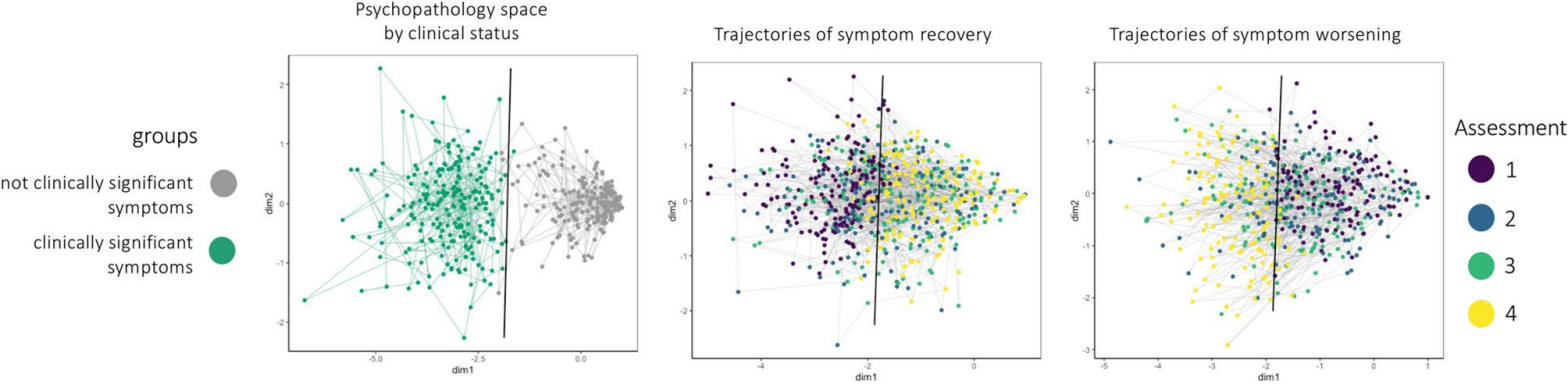
Trajectories through multidimensional space capture changes in CBCL assessment across four years. Left panel: Distinction between adolescents meeting clinical criteria (psychopathology space: green) and those not meeting clinical criteria (non-psychopathology space: grey). A logistic regression decision boundary (black line) delineates the region of space associated with clinically significant symptom profiles, as defined by a CBCL Total Problems T-score ≥ 65. Center panel: Adolescents who began the assessments with clinically significant symptoms and improved over time move from psychopathology space to non-psychopathology space (left → right). Rightpanel: Adolescents who began the assessments without significant symptoms and developed them over time move from non-psychopathology space to psychopathology space (right → left). Note this space was generated from symptoms plus demographic features, not diagnoses.

The HDSS framework enables the visualization of individuals’ trajectories over time through a shared psychopathology space (Fig. 4). This approach allows for simultaneous representation of (1) an individual’s current location in psychopathology space, reflecting their symptom profile (2) longitudinal tracking of symptoms across assessments, and (3) directional change relative to a clinical threshold. Adolescents meeting clinical criteria (CBCL total problems T-score greater than 64) consistently occupy a distinct region of the space, with a logistic regression model identifying a linear decision boundary that effectively separates clinical from non-clinical observations. Transitions across this boundary reflect symptom improvement or worsening, demonstrating that clinical status is reflected in the structure of the space.

### DSM-5 categories and HiTOP dimensions in HDSS

**Fig. 5:**
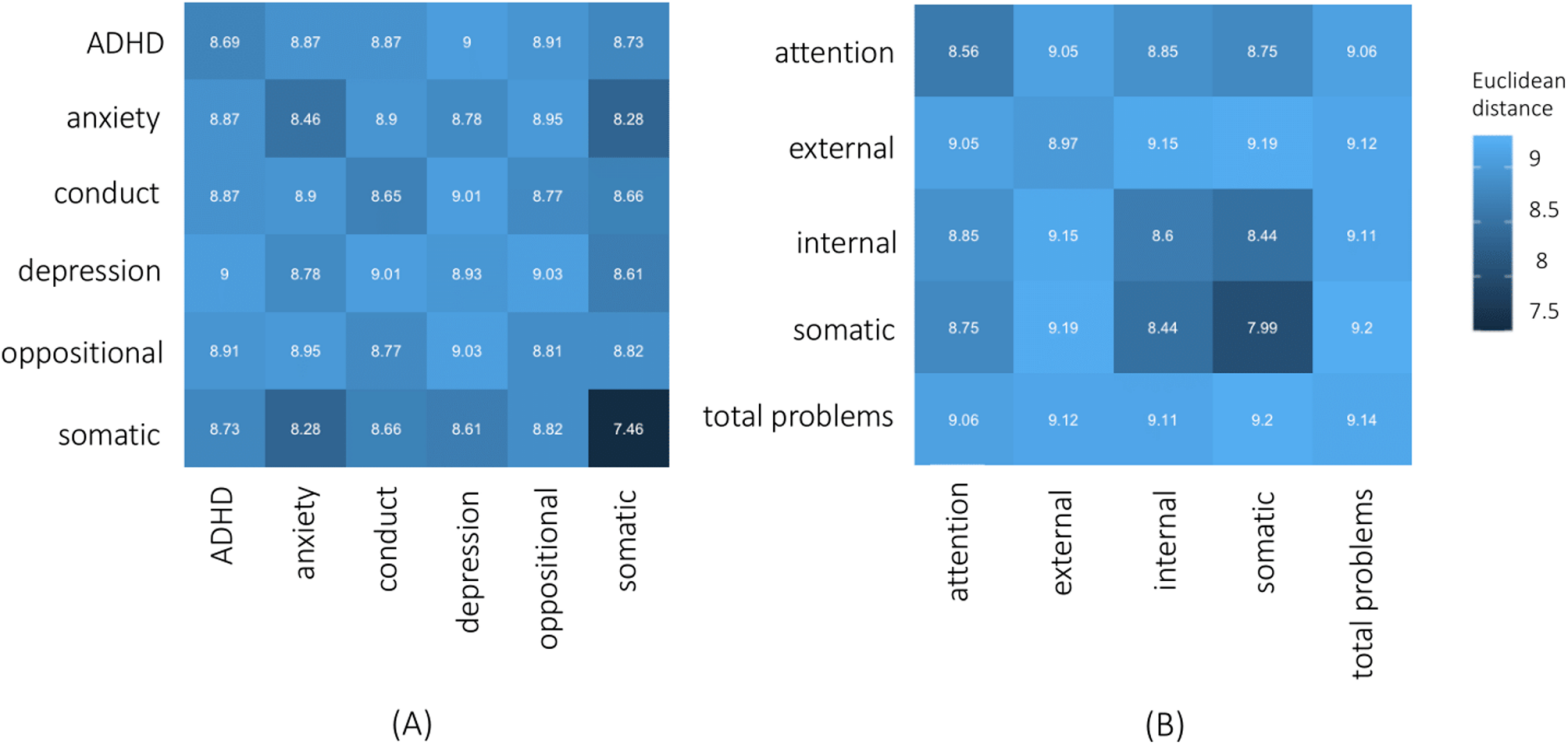
Pairwise matrices of Euclidean distance in High-Dimensional Symptom Space (HDSS) between (off-diagonal) and within (diagonal) clinically significant symptom groups. (**A**) Average distance between individuals meeting criteria for DSM-5 disorders. (**B**) Average distance between individuals with clinically significant scores (CBCL T-score ≥ 65) on HiTOP symptom dimensions. In both panels, the x- and y-axes represent the same set of categories or dimensions. Darker colors indicate greater similarity (i.e., shorter distance).

Figure 5A demonstrates that the distances within disorder categories (M=8.5, SD=0.5) are only minimally lower than those between disorder categories (M=8.8, SD=0.2), indicating no clear pattern of disorder categories being more similar to themselves (Fig. 5A; diagonal from bottom left to top right) than to other disorder categories (Fig. 5A; the off-diagonals).

When we examine the relative similarities of adolescents in MDS based on HiTOP-style dimensions, a similar pattern to DSM-5 categories emerges (Fig. 5B). The pattern of ineffective categorization is replicated, with within-dimension distances (M = 8.7, SD = 0.4) only slightly lower than between-dimension distances (M = 8.9, SD = 0.2). This suggests that HiTOP dimensions, like DSM-5 categories, do not effectively differentiate adolescents’ symptoms in multidimensional space.

### Clinical significance in HDSS

**Fig. 6:**
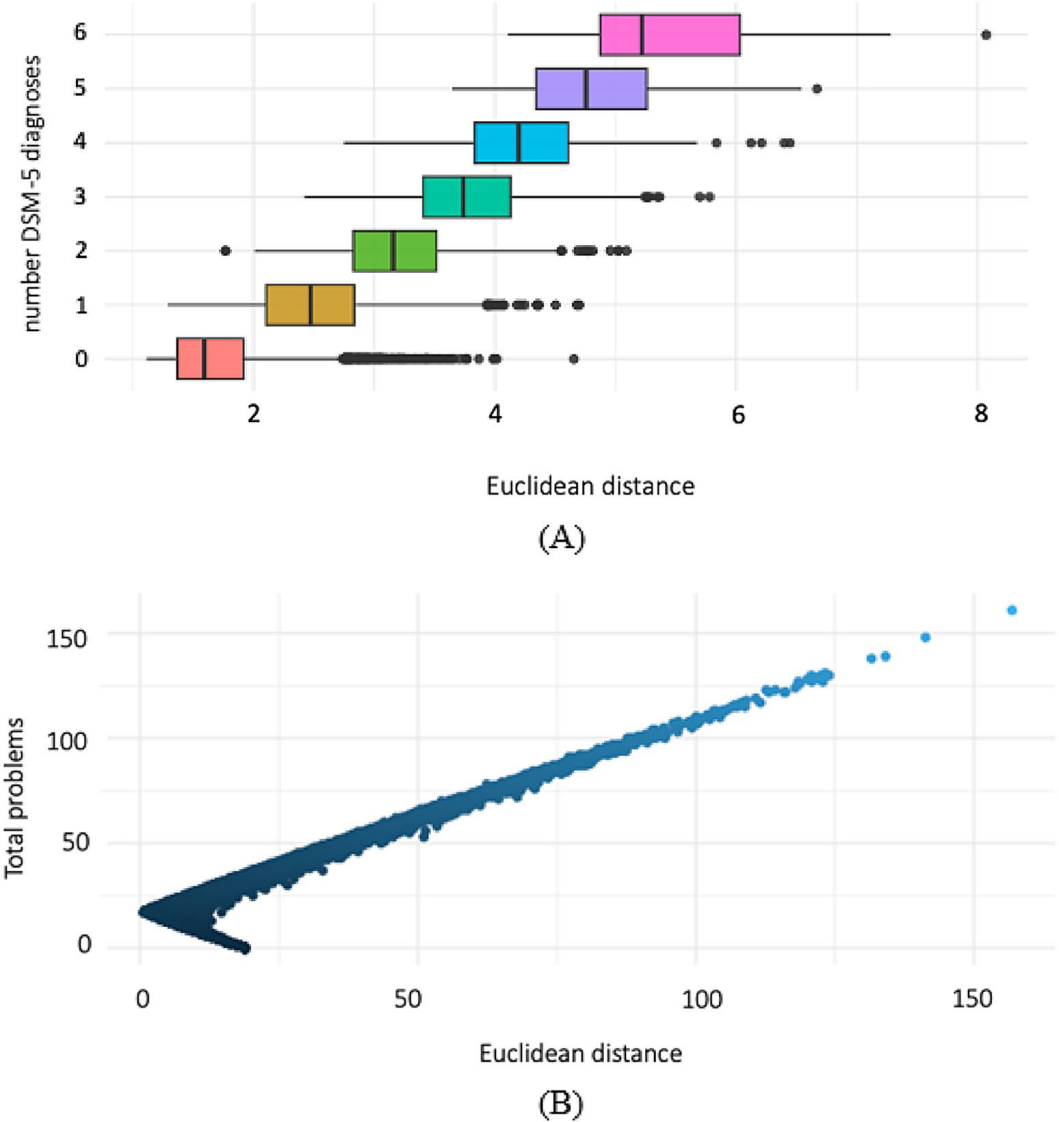
(**A**) Euclidean distance from the CBCL and demographics average vector is associated with the number of DSM-5 diagnoses, indicating that greater distances are associated with an increasing number of clinical diagnoses. (**B**) Euclidean distance from the five-dimensional HiTOP average vector is associated with total problem scores, demonstrating a close relationship between distance in the multidimensional space and the overall severity of symptoms.

The regression model (*p* < 0.001, adj. R² = 0.66, BIC = 29729) shows that as DSM-5 diagnoses increase, so does the Euclidean distance from the average person vector. This suggests that the HDSS model captures clinical severity beyond merely counting diagnoses, as people with no clinical diagnosis can still exhibit high symptom endorsement, comparable to those with multiple diagnoses (Fig. 6a). Distance also scales closely with total symptoms endorsed, as shown by a strong Pearson’s correlation (*r* = 0.74, *p* < 0.001) and a linear regression model (beta = 1.03, t = 212.75, *p* < 0.001), demonstrating the close relationship between distance and symptom severity. However, the two are not perfectly correlated, indicating that this approach offers valuable information about the specific pattern of symptoms (i.e., that two people can have the same number of symptoms but different specific symptoms) that is lost in a summed symptom score (Fig. 6b).

## Discussion

We propose the HDSS model of psychological symptoms as features used to define vectors in multidimensional space. We show that (1) adolescent psychopathology can be successfully modeled using HDSS, (2) unique person-level trajectories through symptom space can be generated from psychopathology symptom features, (3) HDSS captures clinical heterogeneity while still providing useful reference points for pathology (i.e., centroid analyses), and (4) HDSS maintains person-level specificity that is lost in DSM-5 and HiTOP-based models.

The HDSS model offers person-specific quantification of psychopathology symptoms, accommodating individual variability beyond current categorical (e.g., DSM-5) and dimensional (e.g., HiTOP) approaches. As a data analytic method, the HDSS model integrates multimodal data, enabling a mathematical representation of the biopsychosocial model and person-centered care. It has been a challenge to successfully integrate biopsychosocial features into a single model due to the number of predictors allowed in statistical models. Defining a person’s experience in the HDSS model provides a much more accurate quantification of that person (Fig. 4). HDSS provides a flexible, data-driven alternative to stringent classifications, allowing for deduction rather than reduction. The central benefits of a multidimensional approach are that it preserves a person as an individual point in space, rather than being artificially categorized, and allows more precise modeling of psychopathology across people. HDSS is a step toward these goals in two ways: as a method that can be combined with other models and as a model that captures person-level heterogeneity from commonly collected features.

As a method, HDSS can also be combined with other promising methods for modeling psychopathology at the person-level. For example, network approaches provide insights into the complex interconnections of symptoms and experiences in the biopsychosocial processes that underlie mental health^3,32,55,56^. Further, idiographic network models hold great promise for person-level modeling of psychopathology symptoms and their changes over time^57^. While both person-specific approaches, HDSS and idiographic models are complementary approaches that answer different questions: HDSS helps to define psychopathology by comparing among people, and idiographic approaches could then be used to track a specific person’s experience over time. Future work could use HDSS to identify the relevant features to include in a person’s network that then tracks their psychosocial experience and responses to treatment over time.

The HDSS model also captures clinical heterogeneity while providing useful reference points for pathology (i.e., centroid analyses) without imposing subjective categories. Centroid analyses dynamically norm each sample, making each person’s distance from this norm informative, irrespective of diagnostic criteria. This contrasts sharply with DSM-5 categories, where people are treated uniformly based on disorder presence or absence. The result of this process is that, according to DSM-5, the distance within a disorder category should be minimal and the distances between disorder categories should be large, because these are separable experiences. However, Fig. 5 shows no discernable difference in the distances between versus the distances within a diagnostic category. As a result, this immense amount of variability is obscured when categories are used, and the categories themselves do not demonstrate a clear decision boundary (Figs. 3 and 6). This lack of discernible difference in distances is maintained even when dimensional structure is used. However, the distance in space is related to the overall severity in symptoms and number of DSM-5 disorders (Figs. 4 and 6).

In this sense, the distance metric can quantify severity of psychopathology. Mathematically, distance from the average can only increase with the endorsement of more or more severe symptoms and can only decrease with the endorsement of fewer or less severe symptoms. This distance from the average vector is an improvement over *total scores or dimensions*, such as the total problem dimension of the CBCL, because distances in HDSS are paired with a direction. As such, different patterns of symptoms with the same distance from average (i.e., the same total problems total score) can be treated as the different psychological experiences they are because they will be in different parts of space.

Importantly, distance must be interpreted in the context of the data used to create the space. The distance from the centroid represents a distance from normative features in the sample. If, as is the case with the ABCD sample presented, demographic factors are included and the sample is largely White American and female, then distance from the overall average vector (including demographic features) will be greater for anyone not sharing these identities. In this case, distance does not represent pathology. However, if an average vector is created for each combination of demographic data (e.g., an average vector for people who identify as American Indian and male, etc.), and only the vectors of people with those demographics features are compared to generate distances, now the distance metric is not only an index of symptom severity (as determined by the societal standards inherent in the assessments used), but also dynamically normed on multiple features of identity. Average demographic vectors could then be compared to quantify disparity. Similarly, to create more clinically relevant models, dimensions of the space could be weighted based on user need (e.g., clinician judgment that change in a measure is clinically meaningful).

### Clinical applications

The HDSS model also extends theory in the conceptual modeling of psychopathology with quantitative methods that could be clinically implemented. It synthesizes data from assessments, self-report, and clinician intuition into a vector summarizing a person’s biopsychosocially relevant data. As more people are added, the model can identify aberrant experiences, creating a data-driven psychopathology model relevant to the included population. Person-level complexity is maintained in a format that still allows for interpretation. Clinical intakes do not have to be reduced to diagnoses but rather allowed to maintain their nuance and uniqueness.

An issue with current clinical models is that people with significant symptom endorsement often do not meet diagnostic criteria for a specific disorder. These people would not receive treatment given their lack of diagnosis and are therefore missed by the current categorical approach. By contrast, the HDSS model captures heterogeneity in meaningful psychological distress among people who may not meet specific criteria for a discrete disorder (Figs. 3 and 4). Figure 6 demonstrates that as distance from the average vector increases, so does the number of DSM-5 diagnoses, and vice versa. Therefore, distance from the average vector can approximate recovery or progression of a disorder, with decreasing distances indicating recovery and increasing distances indicating progression. A similar pattern is observed when comparing distances to total symptoms endorsed in the HiTOP (i.e., five dimensional) space (Supplementary Fig. 4).

Further, it is possible to use the experiences of people located in a similar space (e.g., shared experience of suicidal behavior) to quantify a threshold of increased risk defined by the space via a hyperplane. The hyperplane boundary can be used as a threshold to predict people’s trajectories toward negative psychosocial outcomes (e.g., suicidal behavior). Such predictions and the components of the vector that are contributing to a person’s trajectory towards a negative outcome can then guide precise interventions to alter the trajectory of that person and avert negative outcomes before they occur. The result of this process could be opportunities to engage mental health interventions at the points at which they are most effective: prior to the development of chronic negative impairment (i.e., the perpetuating stage of psychopathology)^58^. The degree to which such interventions are effective can also be quantified using the HDSS model, as the degree of deflection of the trajectory away from a more severe symptom space.

### Perspectives and future directions

The HDSS model offers several promising future directions, including (1) incorporation of demographic and other cultural information that has been excluded from DSM-5 and HiTOP approaches (see Supplementary Fig. 3), offering a potential avenue to begin to address systemic bias, by accounting for social determinants of health across the spectra influenced by systemic oppression; (2) the creation of more specific spaces to examine subcategories of psychological experience (e.g., PTSD space, chronic pain space); (3) application of modern clustering approaches, such as density based spatial clustering of applications with noise (DBSCAN), to identify data-driven categories of experience for more targeted and efficient interventions; and (4) the prediction of as of yet unreleased ABCD data with symptom space trajectories, including allowing parents and children to exist within the same symptom space, thus capturing the complex interaction of factors responsible for transgenerational psychopathology.

Prediction in particular may be a fruitful future direction. The HDSS model accommodates trajectories and could therefore allow prediction of future behavior. By analyzing a person’s past trajectory in multidimensional space, future points can be predicted, either based on the person’s history or trajectories of similar others. This dual capability provides personalized insights while allowing comparisons that inform treatment targets across people, as seen in Fig. 4. Further, the space can also allow for developmental changes, addressing a key aspect of psychopathology that benefits from predictive modeling^59^. The changes in trajectory over time were notable in just the four years of development captured in this wave of ABCD data.

### Limitations

The HDSS model has limitations that should be considered. First, generalizing from one space to another may be challenging. However, a benefit of the HDSS model is that smaller spaces can be combined based on shared features, allowing for dynamic improvement as new features and data become available. Another limitation is that while centroids can be customized to individual identities, many current datasets confound disparities and normative differences due to biased assessments, such as differential item functioning. This issue could be resolved by comparing functioning to create “healthy” and “pathology” groups in large datasets and by improving assessment instruments. A final limitation is that current modeling approaches cannot accommodate an infinite number of features, known as the curse of dimensionality. When model sizes reach tens of thousands of dimensions, performance begins to depreciate. However, the HDSS model mitigates this concern in two ways: (1) by focusing on prediction within the same sample rather than new samples, and (2) by leveraging rich datasets like ABCD, which do not approach such high dimensionality when the sample size is sufficiently large. Thus, the curse of dimensionality would likely only become an issue if raw neuroimaging data or full genetic sequencing data were introduced into the model. The data used in the current study also has limitations, with the relatively small number of features, use of parent-report alone, and examination in one, albeit large, dataset. Further, our comparison to HiTOP is attenuated by the small number of dimensions used to represent the HiTOP approach. Inclusion of a richer HiTOP structure may better define future iterations of HDSS models.

### Conclusion

Multidimensional modeling of psychopathology has the potential to operationalize precision psychiatric care. A collection of information related to a specific patient can now be collated into a precise quantification of their experience through the HDSS model, providing an “address” of experience. That experience can then be predicted over time, with salient features intervened upon to alter trajectories away from disordered experience and toward well-being. In doing so, the HDSS model quantifies the integrative, multidisciplinary models of psychopathology called for by leaders in clinical science, neuroscience, and implementation science^34^.

## Supplementary Information

Below is the link to the electronic supplementary material.

Supplementary Material 1

## Data Availability

The data used in this study are publicly available from the National Institute of Mental Health’s Data Archive (https://nda.nih.gov/).
