# Supplementary material for: Modeling psychopathology in high-dimensional vector space using the high-dimensional symptom space (HDSS) model can operationalize precision psychiatry in US adolescents
Source: Sci Rep. 2025 Oct 8;15:35084. doi: 10.1038/s41598-025-18975-y (PMC12508039; doi:10.1038/s41598-025-18975-y)
Supplement: Supplementary file 1 — Supplementary Material 1 [file 41598_2025_18975_MOESM1_ESM.pdf]

Modeling psychopathology in high-dimensional vector space using the High-Dimensional Symptom Space (HDSS) model can operationalize precision psychiatry in US adolescents

Marcus G. Wild [1] and Rebecca A. Cutler [2]

[1] VISN 17 Center of Excellence for Research on Returning War Veterans, Waco, TX, USA
[2] University of Texas at Austin, Austin, TX, USA

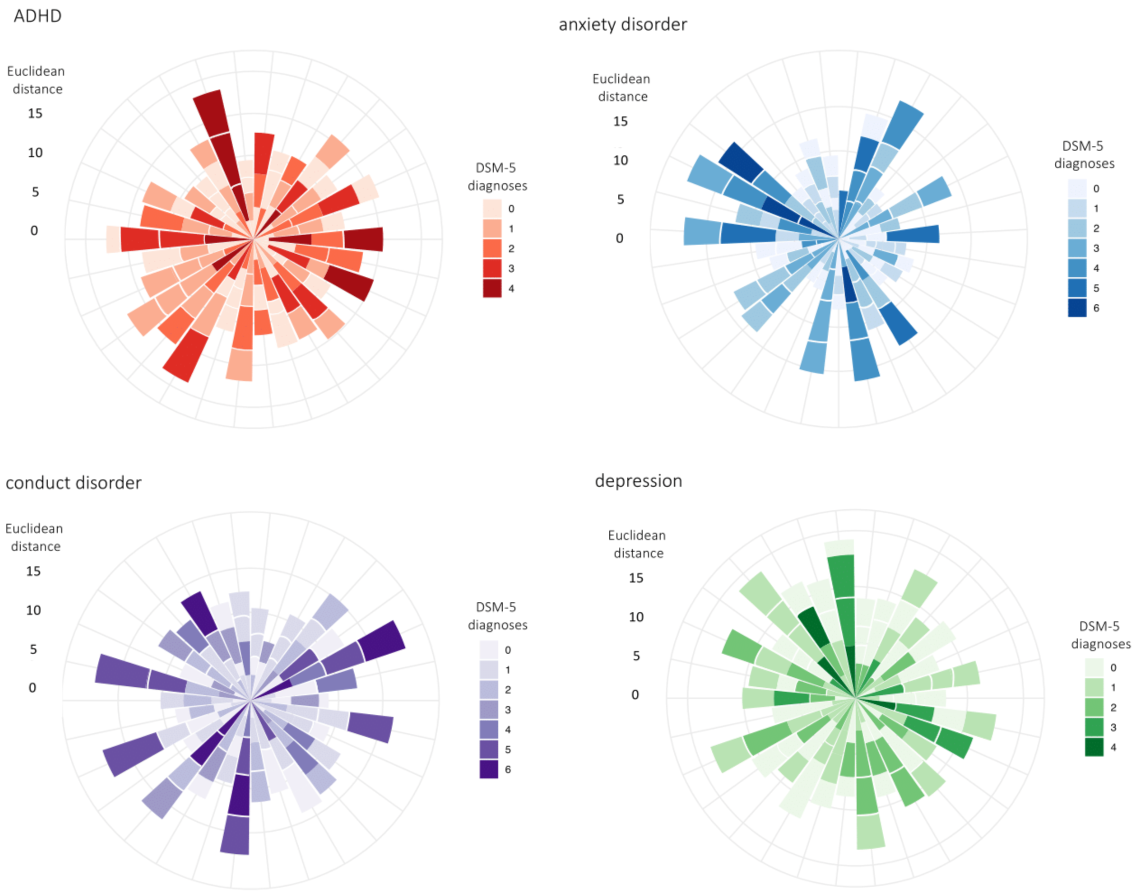

Supplementary Figure 1. Rose plots showing distance from the average person vector for four additional DSM-5 diagnostic groups. Each plot includes 30 randomly selected adolescents who met criteria for the specified diagnosis: ADHD, anxiety disorder, conduct disorder, and depression. Each bar represents one individual, and the four stacked segments reflect their Euclidean distance from the average symptom vector at each of four assessments. Longer segments indicate greater deviation from the average symptom vector. Light-dark color axis indicates the number of DSM-5 diagnoses at each timepoint.

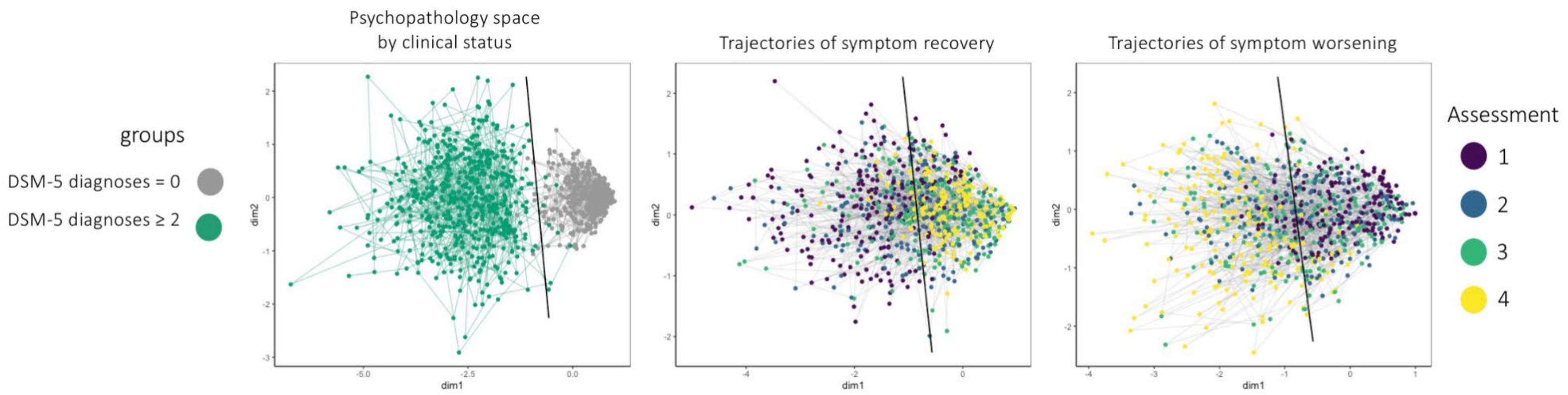

Supplementary Figure 2. Trajectories through High-Dimensional Symptom Space (HDSS) by DSM-5 diagnostic status. Left panel: HDSS representation of adolescents with no DSM-5 diagnoses (grey) and those with ≥2 DSM-5 diagnoses (green). A logistic regression decision boundary separates clinical (DSM-5 diagnoses ≥ 2) and non-clinical groups (DSM-5 diagnoses = 0). Middle panel: Adolescents who began in the clinical group and moved toward the non-clinical region of HDSS over time (recovery trajectories). Right panel: Adolescents who began in the non-clinical group and moved toward the clinical region (worsening trajectories). Point color indicates assessment wave (1–4). Lines connect observations from the same individual across time.

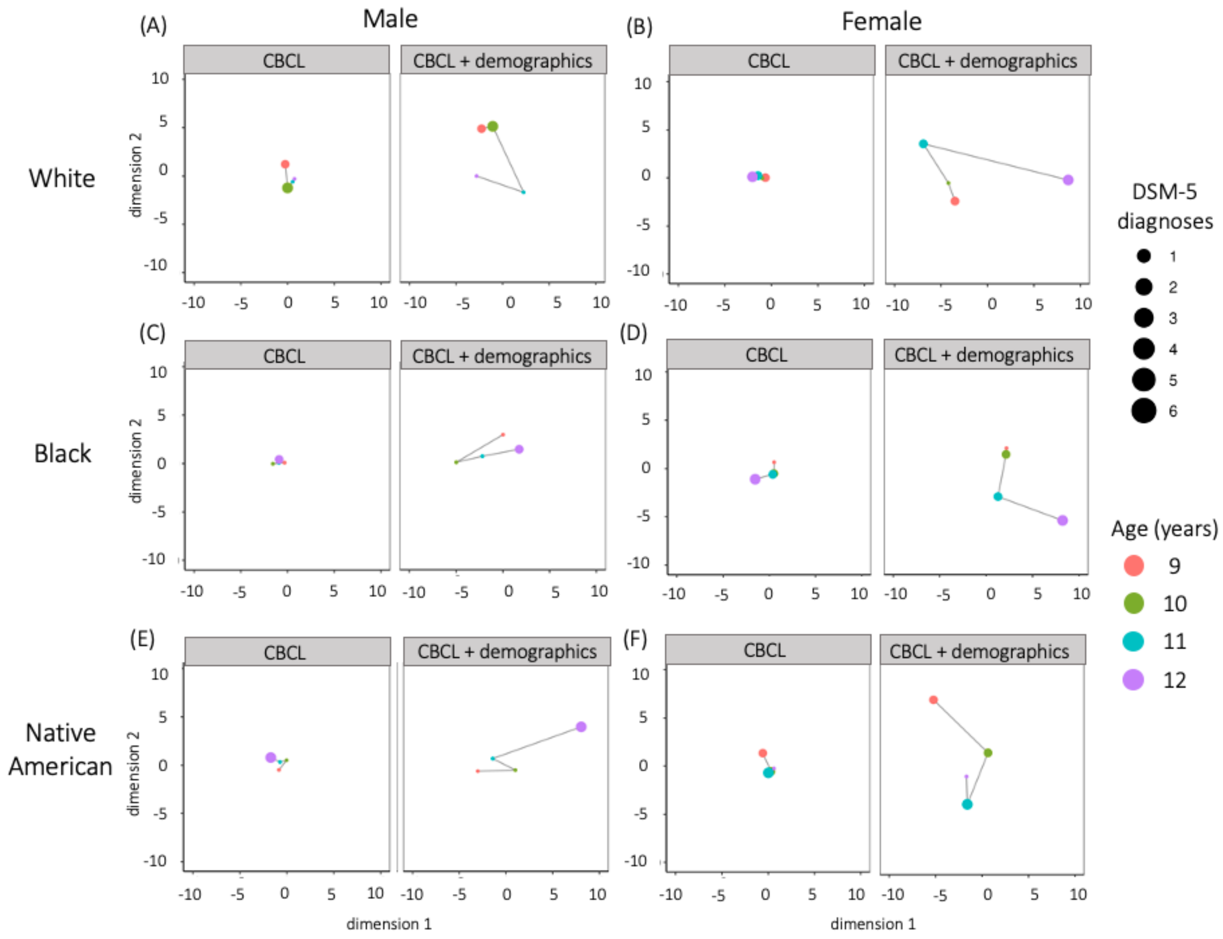

Supplementary Figure 3. Trajectories through multidimensional space capture changes in CBCL assessment across four years. Six example adolescents and how their trajectories are altered if gender (columns) and race (rows) are included as dimensions of the space for (A) White male, (B) White female, (C) Black male (D) Black female (E) Native American Indian male, and (F) Native American Indian female American participants.

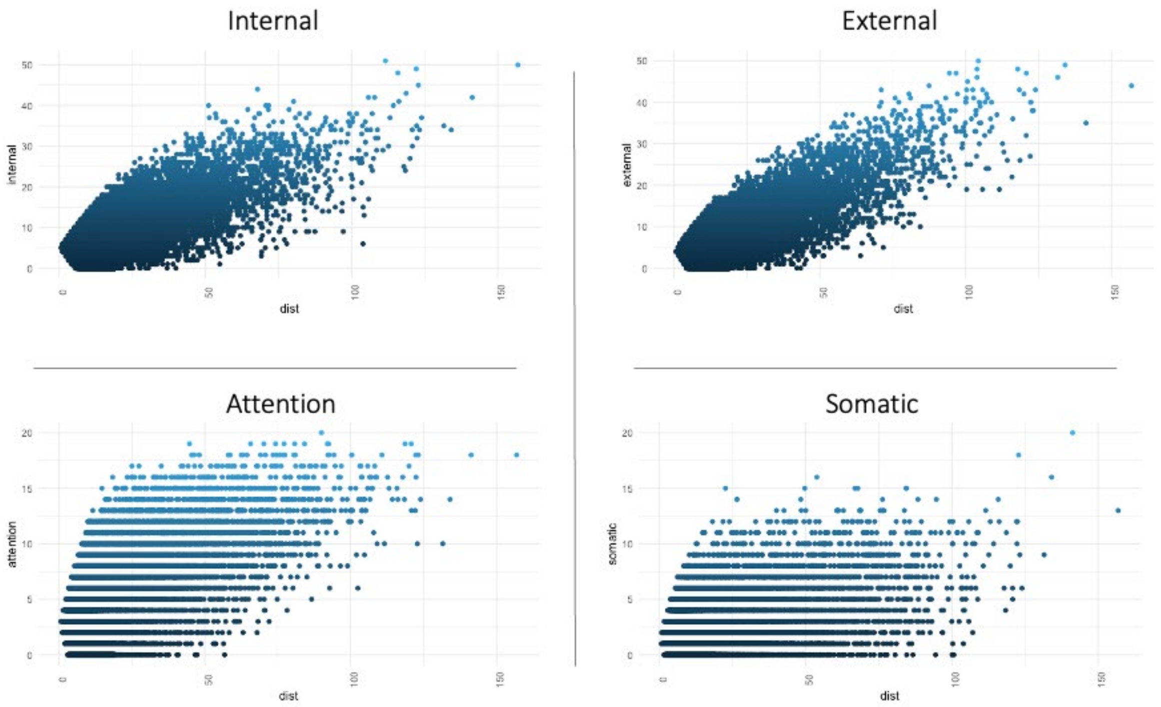

Supplementary Figure 4. Scatter plots illustrating the relationship between the distance from the average vector in the five-dimensional HiTOP space (x-axis) and the number of symptoms endorsed within each psychopathology dimension (y-axis) for internalizing, externalizing, attention, and somatic symptoms. Each plot highlights the positive correlation between distance and symptom count, showing how greater distances are associated with higher symptom endorsements across dimensions.
